# Three-screw versus two-screw fixation of distal fragment in fifth metacarpal neck fractures stabilized with locking plate

**DOI:** 10.1038/s41598-017-12771-z

**Published:** 2017-10-02

**Authors:** Hongyi Zhu, Bingbo Bao, Xianyou Zheng

**Affiliations:** Department of Orthopaedic Surgery, Shanghai Jiaotong University Affiliated Sixth People’s Hospital, Shanghai, 200233 China

## Abstract

Fifth metacarpal neck fracture commonly requires open reduction and internal fixation. Locking plate was widely adopted in the treatment of fifth metacarpal neck fracture as first-line choice for fixation. Patients with fifth metacarpal neck fracture receiving locking plate fixation were included for analysis. Features of internal fixation including number of distal and proximal locking screws, diameter of the screws and usage of lag screws were recorded. Clinical and radiographic outcomes included final volar angulation, grip strength, Michigan Hand Outcomes Questionnaire (MHQ) and range of motion (ROM) of fifth metacarpophalangeal joint. Three-screw fixation was less frequently presented in the group with increased volar angulation (≥30 degrees). Consistently, three-screw fixation of distal fragment could improve the prognosis compared with two-screw fixation (MHQ 95.4 ± 5.1 versus 80.4 ± 12.3, ROM 83.5 ± 7.2 versus 69.6 ± 7.7). In conclusion, the metacarpal head should be fixed by three locking screws instead of two locking screws.

## Introduction

The fifth metacarpal neck fracture accounts for approximately 20% of all fractures in hand^1^. A biomechanical study showed that final volar angulation more than 30 degrees could lead to decreased ROM of fifth metacarpophalangeal joint and functional impairment^2^. A range of surgical techniques have been described for the treatment of fifth metacarpal neck fracture: intramedullary K-wires^3^, transverse K-wires^4^, tension band^5^, locked intramedullary nailing^6^, external fixation^7^, and locking plate fixation^8,9^. A meta-analysis of six studies recommended locking plate as first-line choice of operative fixation^10^.

The secondary displacement in volar angulation was frequently observed after locking plate fixation, highlighting the need for improvement. We therefore showed that locking plate in combination with two crossed K-wires could decrease the secondary displacement in volar angulation compared with fixation with locking plate alone in previous study^11^. However, percutaneous K-wire fixation could also lead to an increase in surficial infection and irrigation of skin^12^. In addition, pin tract infection was also a major complication after K-wire fixation^13^. Thus, it is valuable to find how to improve the locking plate fixation without insertion of K-wires.

Features of internal fixation including number of distal and proximal locking screws, diameter of the screws and usage of lag screws is associated with the strength of fixation and therefore may affect the secondary displacement and prognosis^14^. However, to the best of our knowledge, there was no previous analysis of the association between these features of locking plate fixation and clinical outcomes. As a result, the fixation patterns were mainly based on the surgeons’ experiences and preferences. We hypothesized that specific fixation patterns might have a better outcomes compared with the rest ones. We therefore conducted an observational investigation on previous cases to find the better patterns for locking plate fixation.

## Materials and Methods

The study was approved by the Ethics Committee of Shanghai Jiaotong University Affiliated Sixth People’s Hospital. Informed consents were obtained from all participants and all methods in this study were in accordance with the Declaration of Helsinki. All patients with acute fifth metacarpal neck fractures (≤3 days) treated with open reduction and locking plate fixation from February 2014 to February 2016 were included for analysis. Patients with any of the following criteria were excluded initially: age below 20 or above 60, any injuries on tendons, ligaments, vessels and nerves on the upper limbs, fractures on the upper limbs in addition to fifth metacarpal neck, open fractures, immobilization after surgery. Fifty-nine patients were excluded from analysis due to incompletion of one-year follow-up.

**Figure 1:**
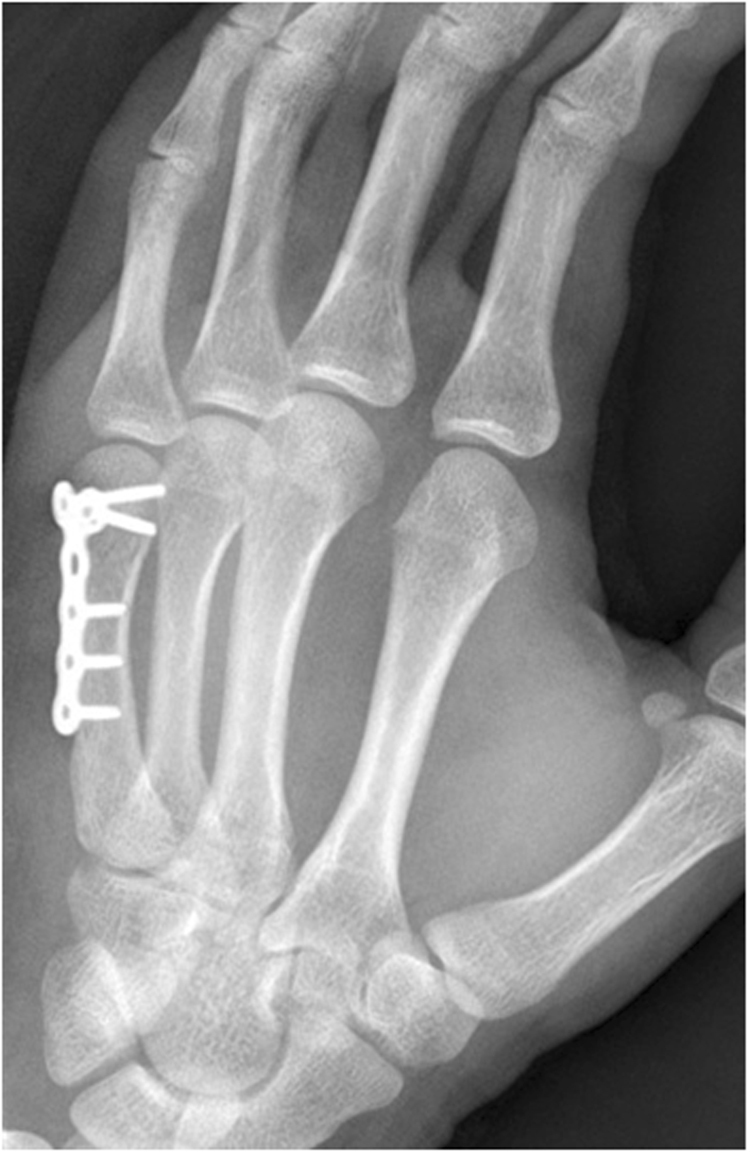
A representative radiograph after locking plate fixation.

All patients were operated through a longitudinal dorsal approach facing the fifth metacarpal axis. T-shaped plates (three distal holes) or Y-shaped (two distal holes) plates were adopted based on the surgeon’s preferences to stabilize the fractures (Fig. 1). The plates in this study were provided by three manufacturers including Acumed (the U.S.), Guci (China) and Kangli (China). All surgeries in this study were conducted by 19 specialists of hand surgery with identical grade. There was no postoperative immobilization, and active mobilization was encouraged immediately after surgery. Patients were then regularly followed up. All patients received routine therapist-guided physical rehabilitation during the initial three months after surgery.

Demographic parameters including age and gender were recorded for each group. Clinical assessments were conducted at 1 year after treatment. Clinical assessments included grip strength, Michigan Hand Outcomes Questionnaire (MHQ)^15^, range of motion (ROM) of fifth metacarpophalangeal joint. Grip strength was measured with the elbow flexed at 90 degree and the forearm in neutral rotation. Values are expressed as percentages of the values of the contralateral hand. The MHQ is a 37-item questionnaire, which is divided into the same distinct 6 subscales: overall hand function, activities of daily living, pain (reversed), work performance, aesthetics, and patient satisfaction. The MHQ total score is obtained by averaging the scores for all 6 subscales. The total score ranges from 0 to 100 with a higher score indicating better hand performance. The ROM of fifth metacarpophalangeal joint was also recorded using a finger goniometer. The volar angulation and axial shortening was measured by the plain radiograph based on methods introduced and advocated in previous reports^16,17^. For the measurement of volar angulation, the distal line was drawn from the mid-medullary point in the distal fragment to the most distal point of the metacarpal head, and the proximal line centrally through the shaft medullary canal, irrespective of where the line hit the basis of the metacarpal. Radiographic parameters were measured immediately and one year after surgery. Radiographic assessments were performed by two authors. Inter-observer correlation coefficients for volar angulation was 0.82. The values of volar angulation were averaged values from two observers. We randomly chose 50 patients from February 2014 to February 2016 with hand injury but no metacarpal fracture. The radiographs of these 50 patients were all taken for medical purposes.

Statistical analysis was performed using SPSS 22.0 software. A *P* value of <0.05 was considered to be statistically significant. Data were presented as mean ± standard deviation. Student’s t test and chi-square test were used to compare numeric and nonnumeric variables respectively.

## Results

Total 234 patients were included for analysis in our study. All of the treating surgeons were specialists of hand surgery. In our series, 177 of 234 cases were male. The average age of this series was 35.9 ± 8.3 (20 to 59). The right hand was affected in 132, the left hand in 102 cases. Statistically, there was no significant difference of demographics between two groups. The average volar angulation immediate after surgery were 8.2 ± 3.1 degrees and increased to 25.8 ± 8.6 degrees one year after surgery. There was no tendon rupture, deep infection and neurological complications in all patients of this study. Superficial infection, tenosynovitis and malunion were the major complications observed in this study.

Since the volar angulation is highly relevant to the prognosis, we first divided patients into two groups based on the volar angulation one year after surgery. We then compared the volar angulation immediately after surgery and found the values were similar between two groups (8.2 ± 2.9 versus 8.1 ± 3.5, *P* = 0.79). We also measured the volar angulation in 50 patients without fifth metacarpal fractures. The average volar angulation in these patients was 7.8 ± 3.2 similar to the immediate value after surgery. We compared the features of internal fixation between two groups and found the number of distal locking screws were associated with the final angulation (Table 1). Three-screw fixation in distal fragment was less frequently presented in cases with increased final angulation. We also compared the clinical outcomes in patients with two or three distal locking screws (Table 2). The MHQ score and ROM in three-screw group were higher than that of two-screw group. The differences in rates of complications were insignificant.

**Table 1:** Demographics and Features of Internal Fixation at One Year after Surgery (Grouped by Volar Angulation).

	≥30 degrees (n = 152)	≥30 degrees (n = 82)	*P* Value
Male	110 (72)	67 (81)	0.114
Female	42 (28)	15 (19)	
Age	35.8 ± 8.1	36.0 ± 8.6	0.813
Dominant hand	85 (56)	45 (55)	0.935
Body mass index	23.0 ± 2.7	23.5 ± 2.8	0.885
Number of distal locking screws			
2	91 (60)	64 (78)	0.005
3	61 (40)	18 (22)	
Number of proximal locking screws			
3	88 (58)	49 (60)	0.301
4	59 (39)	27 (33)	
>4	5 (3)	6 (7)	
Diameter of locking screws			
≤2.2 mm	95 (62)	59 (72)	0.352
>2.2 mm	50 (33)	20 (24)	
Mixed	7 (5)	3 (4)	
Usage of lag screw			
No	114 (75)	61 (74)	0.923
Yes	38 (25)	21 (26)	
Plate manufacturer			
Manufacturer 1	60 (39)	32 (40)	0.950
Manufacturer 2	49 (32)	28 (34)	
Manufacturer 3	43 (29)	22 (26)	

Figures are numbers (percentage).

**Table 2:** Clinical Outcomes at One Year after Surgery (Grouped by Number of Distal Locking Screws).

	Two screws (n = 155)	Three screws (n = 79)	*P* Value
MHQ^#^	80.4 ± 12.3 (78.5–82.4)	95.4 ± 5.1 (94.3–96.5)	0.0001
ROM^#^	69.6 ± 7.7 (68.4–70.8)	83.5 ± 7.2 (81.9–85.1)	0.0001
Grip strength^#^	91.5 ± 8.2 (90.2–92.8)	90.4 ± 8.6 (88.5–92.3)	0.34
Malunion*	11 (7)	2 (3)	0.15
Superficial infection*	22 (14)	16 (20)	0.24
Tenosynovitis*	6 (4)	3 (4)	0.98

^#^Figures are value (95% confidence interval).

^*^Figures are numbers (percentage).

## Discussion

A previous meta-analysis based on six studies recommended locking plate as the first-line choice of operative fixation^10^. However, secondary displacement in volar angulation which limits the recovery of hand functions could be frequently observed after locking plate fixation, highlighting the need for the improvement of the locking plate fixation. In previous study, we showed that locking plate in combination with two crossed K-wires could decrease the secondary displacement in volar angulation compared with fixation with locking plate alone^11^. However, infection is the major challenge for percutaneous K-wire fixation. In this study, we focused on finding how to improve the locking plate fixation without insertion of K-wires.

There was no previous study, to the best of our knowledge, analyzing the association between features of locking plate fixation and clinical outcomes. As a result, the fixation patterns were mainly based on the surgeons’ experiences and preferences. Hence, we conducted an observational analysis of plate fixation features in this study. Our results revealed that the fixation with three distal locking screws could reduce the secondary displacement and improve clinical outcomes. Hence, we recommended that the distal fragment should be fixed by three locking screws in fifth metacarpal neck fracture. To achieve the three-screw fixation of distal fragment, T-shaped plates (three distal holes) should be adopted instead of Y-shaped (two distal holes) plates. Our study revealed that three-screw fixation of distal fragment was better than two-screw fixation. In addition, the efficacy of locking plate in fifth metacarpal neck fracture should be reassessed since the number of distal locking screws were mainly based on the surgeons’ experiences and preferences previously.

Previous study has indeed reported extensor tendon rupture as a severe complication after plate fixation^18^. However, we did not encounter any patients with tendon rupture in our series. One possibility was that the advances in locking plates including improved biomaterials and anatomically pre-contoured designs have tremendously decrease the rate of this complication^14^. A soft-tissue flap to separate the tendon and plate might also be effective in preventing this complication.

There were several limitations of this study. First, given the observational nature of this study, the number of distal locking screws was decided based on the surgeon’s preference instead of randomly. Second, although the clinical outcomes were blinded when the radiographic assessment was done, the blinding of internal fixation features was impossible. Since all treating surgeons are specialists in hand surgery, personal experiences in clinical practices might cause potential bias on the measurement.
